# Novel rapid intraoperative qualitative tumor detection by a residual convolutional neural network using label-free stimulated Raman scattering microscopy

**DOI:** 10.1186/s40478-022-01411-x

**Published:** 2022-08-06

**Authors:** David Reinecke, Niklas von Spreckelsen, Christian Mawrin, Adrian Ion-Margineanu, Gina Fürtjes, Stephanie T. Jünger, Florian Khalid, Christian W. Freudiger, Marco Timmer, Maximilian I. Ruge, Roland Goldbrunner, Volker Neuschmelting

**Affiliations:** 1 Department of General Neurosurgery, Center for Neurosurgery, Faculty of Medicine and University Hospital Cologne, University of Cologne, Cologne, Germany; 2 Department of Neuropathology, Otto-von-Guericke University, Magdeburg, Germany; 3 Invenio Imaging Inc., Santa Clara, CA USA; 4 Centre for Integrated Oncology, Faculty of Medicine and University Hospital Cologne, University of Cologne, Cologne, Germany; 5 Department of Stereotactic and Functional Neurosurgery, Center for Neurosurgery, Faculty of Medicine and University Hospital Cologne, University of Cologne, Cologne, Germany; 6 Center for Behavioral Brain Sciences (CBBS), Otto-von-Guericke University, Magdeburg, Germany

**Keywords:** Brain tumor, Tissue detection, Neurosurgery, Stimulated Raman histology, Deep learning, Artificial intelligence

## Abstract

Determining the presence of tumor in biopsies and the decision-making during resections is often dependent on intraoperative rapid frozen-section histopathology. Recently, stimulated Raman scattering microscopy has been introduced to rapidly generate digital hematoxylin-and-eosin-stained-like images (stimulated Raman histology) for intraoperative analysis. To enable intraoperative prediction of tumor presence, we aimed to develop a new deep residual convolutional neural network in an automated pipeline and tested its validity. In a monocentric prospective clinical study with 94 patients undergoing biopsy, brain or spinal tumor resection, Stimulated Raman histology images of intraoperative tissue samples were obtained using a fiber-laser-based stimulated Raman scattering microscope. A residual network was established and trained in ResNetV50 to predict three classes for each image: (1) tumor, (2) non-tumor, and (3) low-quality. The residual network was validated on images obtained in three small random areas within the tissue samples and were blindly independently reviewed by a neuropathologist as ground truth. 402 images derived from 132 tissue samples were analyzed representing the entire spectrum of neurooncological surgery. The automated workflow took in a mean of 240 s per case, and the residual network correctly classified tumor (305/326), non-tumorous tissue (49/67), and low-quality (6/9) images with an inter-rater agreement of 89.6% (κ = 0.671). An excellent internal consistency was found among the random areas with 90.2% (Cα = 0.942) accuracy. In conclusion, the novel stimulated Raman histology-based residual network can reliably detect the microscopic presence of tumor and differentiate from non-tumorous brain tissue in resection and biopsy samples within 4 min and may pave a promising way for an alternative rapid intraoperative histopathological decision-making tool.

**Supplementary Information:**

The online version contains supplementary material available at 10.1186/s40478-022-01411-x.

## Introduction

Rapid intraoperative histopathological analysis of fresh frozen tumor tissue is an essential tool for biopsy and surgical resection control [1]. This is based on a time-consuming and costly intraoperative pathology workflow for intraoperative decision-making that often leads to prolonged surgery times with the patient kept in anesthesia waiting for the pathological diagnosis [2, 3]. In addition, not every hospital providing surgeries in patients with brain tumors holds a (neuro)pathology unit available for rapid fresh frozen diagnostics and even if, it is usually only available during normal working hours. Recently, a fully automated label-free optical laser system creating digital hematoxylin-and-eosin (H&E)-like images called stimulated Raman histology (SRH) has been introduced as an alternative to fresh frozen conventional histopathological section diagnostics [4–8]. In brief, SRH is an optical digital molecular microscopy technique that exploits the intrinsic optical properties of biological macromolecules, such as lipids, proteins, and ribonucleic acids, and converts them into digital H&E-like images within 3–4 min. The SRH images are then to be reviewed by a neuropathologist or available for analyses of a machine learning algorithm. The combination of SRH and the use of machine-learning techniques for analysis in a semi-automated workflow has already been explored in some proof-of-concepts investigations [8, 9]. Using convolutional neural networks and the associated evaluation of SRH images, various neurosurgical tumor entities can already be reliably detected [4, 5]. This possibility provides the neurosurgeon during the surgical resection with important information about the tumor entity and glial malignancy to tailor the surgery accordingly. However, the recently introduced CNN to determine the tumor entity and malignancy in the case of gliomas is especially limited in reliably detecting tumor tissue in the tumor margins for resection extent and biopsy control. This study aimed to develop a novel SRH-coupled deep residual CNN for qualitative brain tumor detection independent of entity and dignity and test its reliability to distinguish between tumor and non-tumor brain tissue in a semi-automated clinical intraoperative workflow.

## Materials and methods

In a prospective single-center study design patients were recruited in 2021 after they were indicated for surgery or stereotactic biopsy based on decisions of an interdisciplinary neuro-oncological tumor board panel. This study was reviewed and approved by the local ethics committee (Nr. 21-1238). All patients gave their written informed consent for the scientific use of their data according to European law. Inclusion criteria comprised: (1) Suspected tumor lesions of the central nervous system, (2) aged over 18 years, and (3) the patient is willing and able to give informed consent for participation. Exclusion criteria comprised: (1) Patient does not agree to participate in the study or (2) unable to or unwilling to give informed consent. Specimen samples were excluded if the collected specimen was inadequate, e.g. broken slide or specimen sample size under 1.7 mm diameter for SRH imaging. All specimen samples obtained were imaged immediately after collection by the intraoperative label-free fiber-laser-based SRS microscope and evaluated by the CNN as the output class (1) tumor, (2) non-tumor, or (3) low quality. An independent neuropathologist reviewed all generated SRH images as a control arm.

### Specimen collection and intraoperative SRH imaging method

Tissue samples were intraoperatively obtained from the lesion itself in surgery and stereotactic biopsies as well as from the approach area in surgery. These samples did not interfere with or differ from the regular tumor sampling sent to (neuro)pathology to enable histopathological diagnosis. For virtual imaging of fresh specimens, a small unprocessed specimen sample (3–4 mm in size) was squeezed onto a glass slide with the help of the cover glass and imaged by a clinical, fiber-laser based stimulated Raman scattering microscope (Invenio Imaging Inc, Santa Clara, CA, USA) [8]. In brief, four main components are used here: (1) a fiber-coupled microscope, (2) a dual-wavelength fiber-laser module, (3) a laser and microscope control module, and (4) a computer for data displaying, processing, and application of a CNN. Spectral Raman differences and concentration cause lipids and proteins to reflect differently, creating contrast. Tissue samples were imaged at two Raman shift wavenumbers, 2845 cm^−1^ (CH_2_/lipid channel) and 2940 cm^−1^ (CH_3_/protein and ribonucleic acids). Via another process, a three-channel image was created after image subtraction, resulting in a digital H&E-like stained 3.06 mm^2^ image for intraoperative and pathological view, which is referred to as Stimulated Raman Histology (SRH).

### Machine learning algorithm

A residual convolutional neural network (CNN) architecture named ResNet50v2 was used for image classification on 300 × 300 pixel SRH image patches. The final layers in the network were modified for classification into three classes: (1) tumor, (2) non-tumor, and (3) low quality. The network was trained on a separate previously acquired and annotated dataset of 570 whole-slide SRH images representing the full spectrum of neurooncological surgery that resulted in 1.2 million labeled patches after patch extraction using 300 × 300 pixels sliding window technique with a step of 100 pixels in both x- and y-direction. The CNN training was done on 90% of the training data set, while preliminary validation of the CNN after each epoch was done on 10% of the training dataset. The imbalance of the classes was taken into account by using the inverse class frequencies as weights for the categorical cross-entropy loss of the CNN. The training was stopped after the preliminary validation accuracy exceeded 95% and the loss was lower than 0.10.

**Fig. 1:**
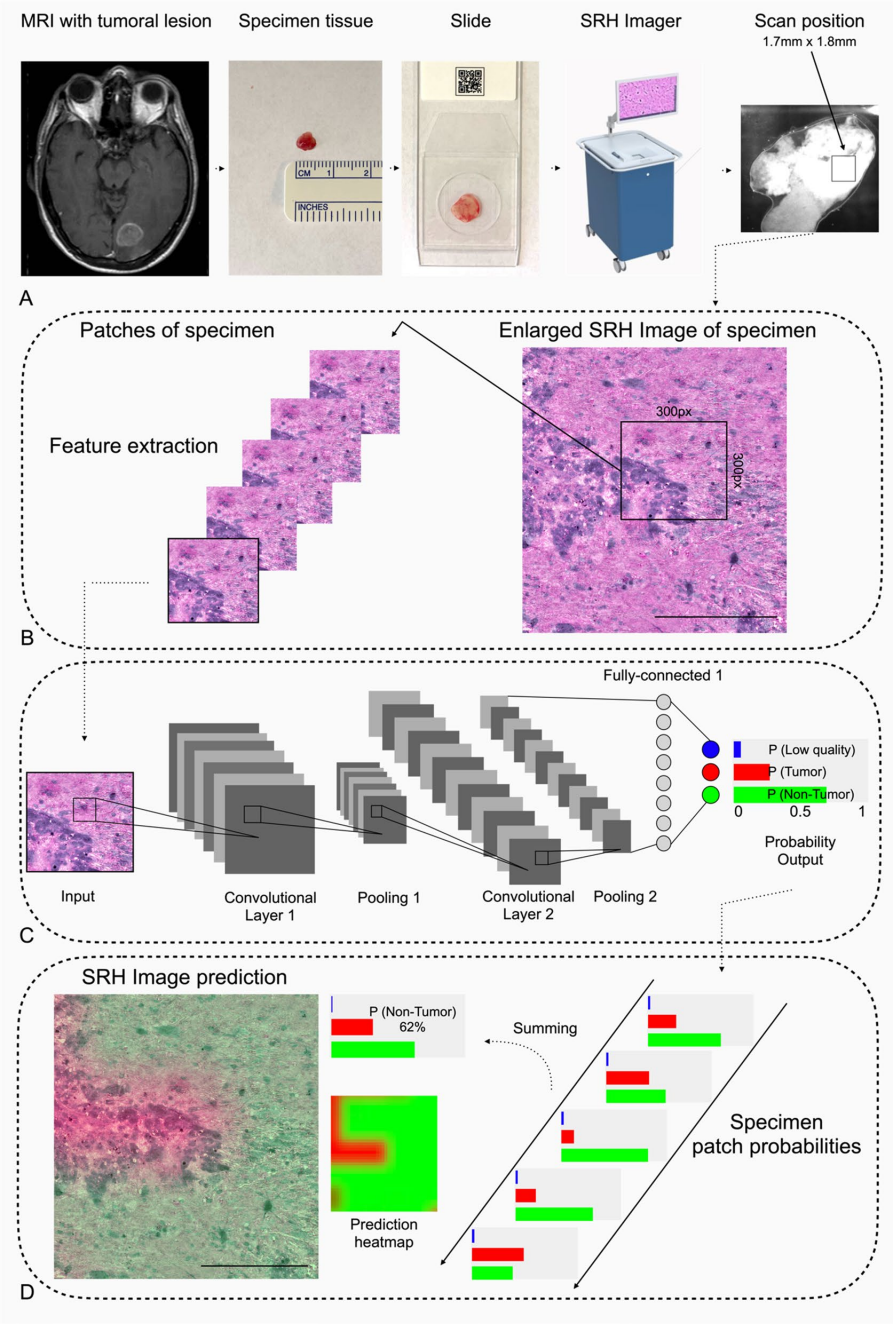
Demonstration of the semi-automated workflow for SRH image analysis and CNN prediction of tumor and non-tumor tissue. **A** A squashed unprocessed tumor margin sample acquired by the surgeon of non-small cell lung cancer brain metastasis to test for residual tumor remnants in the resection bed is analyzed in the intraoperative SRH imager. **B** A digital H&E-like image (SRH) is created. After generating SRH patches (300 × 300-pixel) using a sliding window technique, each patch undergoes a residual CNN algorithm. **C** The final softmax layer outputs a categorical probability prediction with distribution over three categories: (I) tumor, (II) non-tumor, and (III) low quality. After that, another algorithm is applied for the patch-level prediction probabilities and outputs a single probability for each SRH image after summing. A semantic segmentation technique that overlays CNN prediction heatmaps was also developed and applied to facilitate the qualitative identification of regions with tumor, non-tumor, and low quality. **D** Transparency CNN prediction heatmaps were RGB color-coded (red = tumor, green = non-tumor, blue = low quality) and overlaid on the SRH image to provide identification and differentiation for surgeons and neuropathologist beside prediction probabilities. Scale bars = 100 μm

The CNN was re-trained a second and a third time with different random seeds on the same external dataset for reliability and ROC analysis (see Fig. 1).

### CNN Segmentation and probability heatmap of SRH Images

**Fig. 2:**
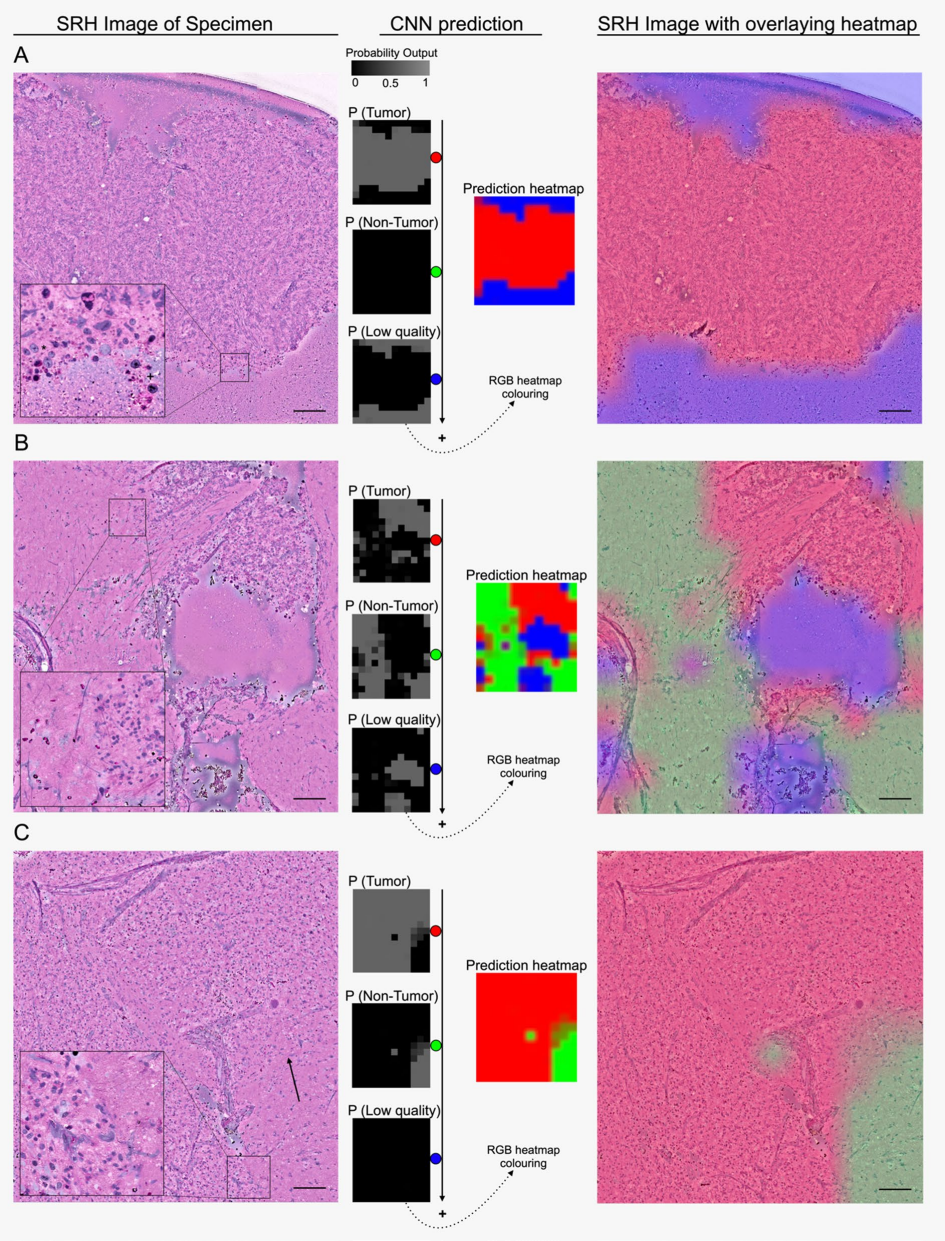
Stepwise semantic segmentation of SRH images for regions with tumor, non-tumor, and low quality. SRH images on the left side are shown before segmentation. In the middle probability, heatmaps are demonstrated for each output P (tumor, non-tumor, low quality). Using a sliding window algorithm, smaller parts in the SRH images created a probability distribution for each output. It is a function of neighboring overlapping patch predictions to generate a smoother overall heatmap after summing each part of the SRH images. Each heatmap is RGB color-coded as an overlay on the SRH image. Demonstration of prediction heatmap (**A**) with tumor (red) and low quality (blue) regions out of a Non-Hodgkin lymphoma specimen (* and + with atypical cell components), **B** with corresponding outputs classes from a non-small cell lung cancer brain metastasis specimen, **C** as well as only tumor (red) and non-tumor (green) prediction from an IDH-wildtype glioblastoma specimen (the arrow demonstrates the infiltrative tumor character). Scale bars = 100 μm

**Fig. 3:**
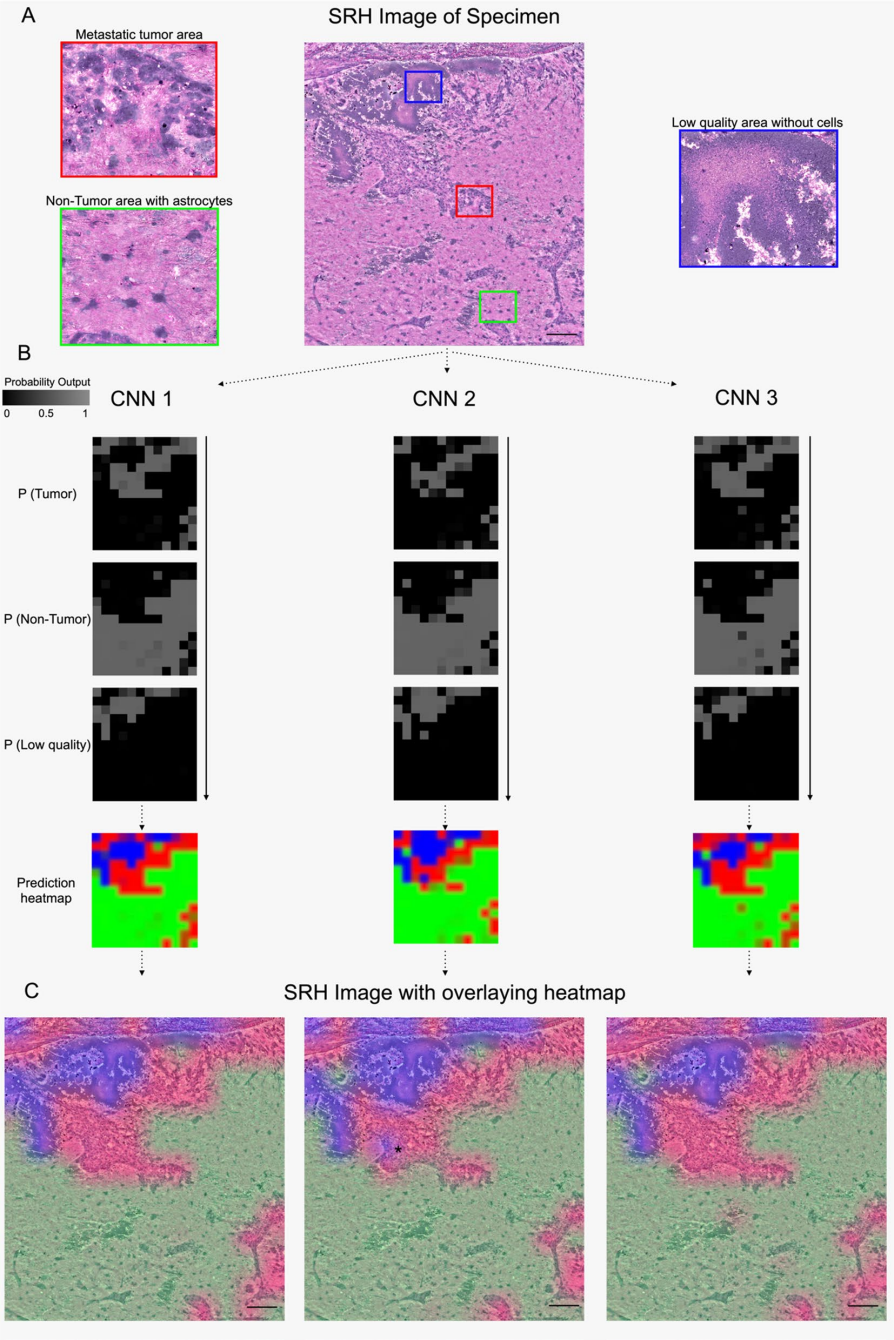
Step by step segmentation by analyzing patch-pixels. **A** Examples of SRH patches with metastatic tumor area (red box), a non-tumor area with reactive astrocytes (green box) and low-quality area without cells (blue box), SRH from a cervical squamous cell carcinoma brain metastasis. **B** Pixel-level probability heatmaps of each output class after patch-passing through all three residual CNNs in comparison. Small differences in classification at the patch size level did not affect the overall prediction output for the whole SRH image. See the left side of the asterisk (*) in the second CNN model. **C** The overall probability heatmaps after summing all patch predictions and mapped as a semi-transparent overlay to assist surgeon and neuropathologist for SRH image interpretation in addition to residual CNNs predictions. Scale bars = 100 μm

To run the CNNs on whole-slide SRH images, testing patches were extracted using a 300 × 300-pixels sliding window with a step size of 300-pixels in both x- and y-direction. In this way, there were no overlapping patches in testing. Patches that had low quality as top-1 class were excluded from the analysis of the final whole-slide prediction, thus only “high-quality” patches probabilities were averaged over the whole-slide image for the final prediction. Heatmaps for whole-slide SRH images (e.g. 3600 × 3900 true image pixels) were created by interpolating the images containing predictions at the patch level (e.g. 12 × 13 patch prediction pixels containing class probabilities). The final probability heatmap of the presence of tumor (red), non-tumor (green), and low-quality (blue) is coded on top of the SRH image as a semi-transparent overlay and could assist the user in interpreting the SRH images by color-coded class probabilities (see Figs. 2, 3).

### Neuropathological validation

The SRH images analyzed by CNNs were reviewed as a high-resolution PNG file with 3600 × 3900 pixels by one board-certified independent neuropathologist, blinded to the CNN results and clinical data, who analyzed, and classified into the three diagnostic classes, (1) tumor, (2) non-tumor, and (3) low quality by a binary evaluation pattern. The predictions of the CNNs and the independent neuropathologist were compared by review on a single image-by-image basis. The highest probability prediction value of the corresponding output class from the CNNs was chosen and thus converted into a binary form to enable the direct comparison to the independent blinded neuropathologist’s evaluation.

### Statistical analysis

Statistical analysis was performed using SPSS Statistics Version 28 (IBM, Chicago IL). For descriptive statistics, continuous values are given in mean with standard deviation, and ordinal and categorical variables are stated in counts and percentages. Internal consistency (IC) of random areas within a specimen sample was calculated using Cronbach's alpha (*C* _*α*_). The inter-rater reliability (three raters) of the probability values between the three trained CNNs was calculated by the intraclass correlation coefficients (ICC) (single-measurement, absolute-agreement, 2-way mixed-effects model). The inter-rater reliability (two raters) between CNNs and neuropathologist was calculated using Cohen´s Kappa (κ). The diagnostic quality of the trained and applied CNNs was validated by the Receiver operating characteristic (ROC) analysis. Values > 0.8 were considered as excellent and > 0.9 as outstanding [10]. As a non-parametric statistic test, Kendall-W-Test was used for significance proofing between CNNs probability values. P-values below 0.05 were considered statistically significant.

## Results

### Demographics and baseline characteristics

We included 94 brain tumor patients, 49 (52.1.%) of them were female. The type of procedure to obtain specimen samples comprised surgical resections in 77 (81.3%) and stereotactic biopsies in 17 (18.1%) patients.

The final histopathological diagnosis was benign to malignant gliomas in 27 (28.7%), various carcinoma and melanoma metastases in 26 (26.6%), benign to anaplastic meningioma in 13 (13.8%), pituitary adenoma in 6 (6.4%), craniopharyngioma in 2 (2.1%), ependymoma in 2 (2.1%), lymphoma in 7 (7.4%), medulloblastoma in 2 (2.1%) patients, other entities such as epidermoid-cyst, ganglioneurinoma, hemangioblastoma, lipoma, rathke cleft-cyst, schwannoma in 6 (6.4%) patients, and gliosis in 3 (3.2%) patients by stereotactic biopsy.

A total of 226 specimen samples were collected for SRH imaging (range 1–8 specimens per patient) resulting in a total of 592 SRH images that were generated (range 1–4 different SRH images per specimen sample). A minimum of three different random areas (A, B, C) per slide were scanned in 132 specimens (58.4%) resulting in 402 SRH images in total for statistical analyses. In three cases of the 402 SRH images, one of the three random areas showed a 100% probability value of low quality, so a fourth random area (D) was obtained for SRH image analysis.

Seventeen (7.5%) microscopic specimen samples resulting in 34 (5.7%) SRH images were obtained as negative controls (healthy macroscopic brain tissue), without contact to a tumor margin, derived from necessary corticotomy in the approach pathway in cases of subcortical located lesions.

Specimen samples from the tumor margins were not obtained from meningiomas, schwannomas, or other extra-axial lesions, but only from malignant intra-axial tumors to test the CNN algorithm reported here.

### Evaluation of CNN models and prediction probabilities

Three CNN models were trained and tested with different random seeds on the same dataset for analysis. Examination and evaluation demonstrated a tumor mean probability of 73.9 (± 33.2) by the first CNN, 76.9 (± 35.0) by the second CNN, and 76.5 (± 33.7) by the third CNN. The interrater analysis of the probability values between the three CNN showed excellent reliability with an ICC value of 0.962 (99% CI 0.953–0.969).

The mean probability for non-tumor was 18.9 (± 33.1) for the first CNN, 18.0 (± 32.7) for the second, and 18.2 (± 31.6) for the third. The ICC was found likewise excellent with a value of 0.977 (99% CI 0.973–0.981) between the three CNNs.

The mean probability for the low-quality output class was 7.2 (± 15.2) for the first, 5.1 (± 16.4) for the second, and 5.3 (± 15.7) for the third CNN. The ICC for the low-quality output class between the CNNs showed excellent interrater reliability of 0.914 (99% CI 0.895–0.929) (further information see Table 2).

In the qualitative image inspection per viewing, some locations of the analyzed SRH images on patch size level showed some differences in the corresponding patch prediction output between the three CNNs but did not affect the overall prediction output of each SRH image. There were no statistically significant differences found between the three CNNs probability predictions regarding the evaluation of tumor tissue (p = 0.231), non-tumor (p = 0.052), and low quality (p = 0.423) SRH images (see Table 1; Fig. 3).

**Table 1:** Comparison of prediction probabilities of the three trained residual convolutional neural networks (CNN) in mean and standard deviation, and Intraclass correlation coefficient (ICC)

SRH images (n = 402) mean probability (± SD)	CNN 1	CNN 2	CNN 3	Reliability (ICC)
*All*				
Tumor	73.9 (± 33.2)	76.9 (± 35.0)	76.5 (± 33.7)	0.962 (99% CI 0.953–0.969)
non-tumor	18.9 (± 33.1)	18.0 (± 32.7)	18.2 (± 31.6)	0.977 (99% CI 0.973–0.981)
Low quality	7.2 (± 15.2)	5.1 (± 16.4)	5.3 (± 15.7)	0.914 (99% CI 0.895–0.929)

### Diagnosis classification by the CNN models and neuropathologist

The neuropathologist established it as the ground truth, considering the majority area of the SRH image for the corresponding annotation, thus identifying 462 (78.0%) SRH images as tumor, 113 (19.1%) as non-tumor (including 34 SRH image negative controls from the surgical access, and tumor negative SRH images derived from tumor margins), and 17 (2.9%) as of low quality.

If one of the three diagnostic classes contained the highest probability prediction value, this class was chosen as the final diagnosis. After summation, the first CNN identified 459 (77.5%) SRH images as a tumor, 115 (19.4%) as non-tumor, and 18 (3%) as low quality.

The second CNN evaluated 456 (77%) SRH images as a tumor, 117 (19.8%) as non-tumor, and 19 (3.2%) as low quality.

The third CNN classified 469 (79.2%) SRH images as a tumor, 110 (18.6%) as non-tumor, and 13 (2.2%) as low quality (see Table 2; Additional file 1: Table S3).

**Table 2:** Overall distribution of the diagnostic classes from the residual CNNs and the independent neuropathologist with the inter-rater agreement between CNNs correct predictions and the neuropathologist (NP)

		NP	CNN 1	CNN 2	CNN 3
SRH images	n = 592				
(All)	100%				
Tumor		462 (78.0%)	459 (77.5%)	456 (77%)	469 (79.2%)
Non-tumor		113 (19.1%)	115 (19.4%)	117 (19.8%)	110 (18.6%)
Low quality		17 (2.9%)	18 (3%)	19 (3.2%)	13 (2.2%)
SRH images (three random areas)	n = 402				
		(Ground truth)			
Tumor		326 (100%)	297 (91.1%)	298 (91.4%)	305 (95.0%)
Non-tumor		67 (100%)	46 (68.7%)	48 (71.6%)	49 (73.1%)
Low quality		9 (100%)	5 (55.6%)	5 (55.6%)	6 (66.7%)

### Diagnostic quality of CNN models

Due to the sensitivity and specificity of ROC analysis, a comparable diagnostic quality could be reached in reference to the independent neuropathological evaluation. In detail, the ROC-AUC of the first CNN was demonstrated as excellent with 0.888 (99% CI 0.838–0.938) for tumor, 0.862 (99% CI 0.789–0.934) for non-tumor, and 0.837 (99% CI 0.576–1.097) for low-quality SRH images.

The ROC-AUC in the second CNN was excellent with 0.895 (99% CI 0.843–0.948) for tumor, 0.879 (99% CI 0.810–0.947) for non-tumor, and outstanding with 0.902 (99% CI 0.751–1.053) for low-quality SRH images.

**Fig. 4:**
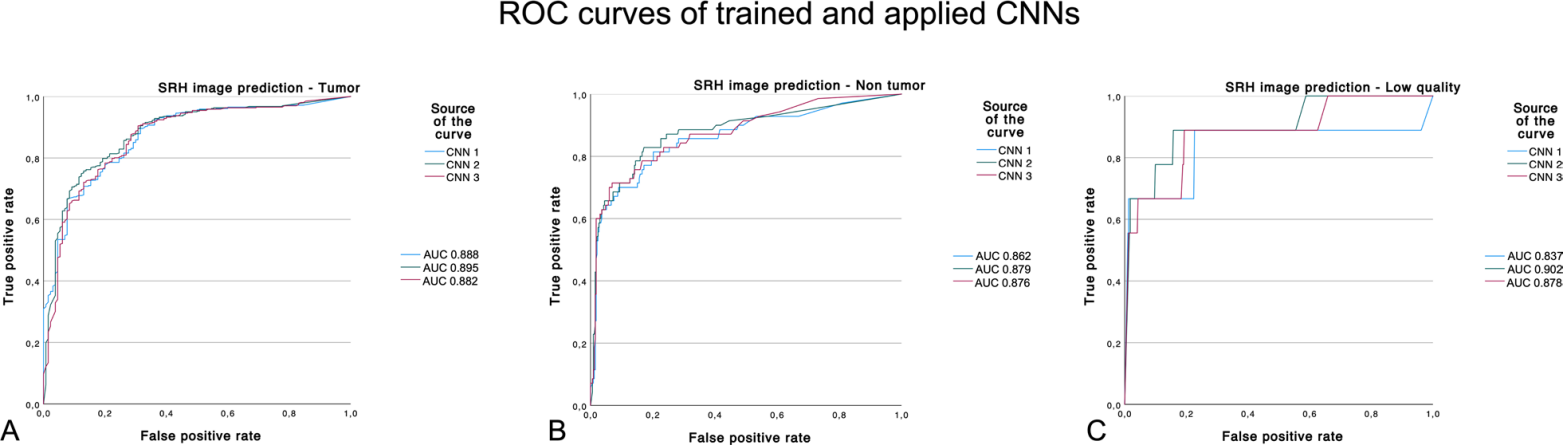
SRH image prediction ROC curves of the three trained and applied CNNs for **A** tumor, **B** non-tumor and **C** low quality tissue, ROC-AUC were similar across each CNN and are showing excellent diagnostic quality in accordance with the independent neuropathological review as ground truth

The ROC-AUC of the third CNN was excellent with 0.882 (99% CI 0.826–0.938) for tumor, 0.876 (99% CI 0.810–0.941) for non-tumor, and 0.878 (99% CI 0.707–1.050) for low-quality SRH images (see Fig. 4).

### Agreement between CNN models and neuropathologist

The lowest inter-rater agreement was found between the first CNN and neuropathological assessment in 86.5% SRH images with a moderate Cohen's Kappa (κ = 0.572).

Compared to the neuropathological evaluation, the second CNN showed an inter-rater consistency in 87.3% SRH images with a substantial Cohen’s Kappa (κ = 0.607).

The best Inter-rater agreement was found between the neuropathological evaluation and the evaluation of the third retrained CNN with 89.6% out of 402 SRH images with correct prediction and differentiation of tumor and non-tumor tissue. There was a substantial Cohen's Kappa (κ = 0.671) (see Table 1).

### Internal consistency analysis

After analysis of the inter-rater agreement between CNNs and neuropathologist, the internal consistency of the three random area SRH images within the same slide (n = 132) was investigated.

**Fig. 5:**
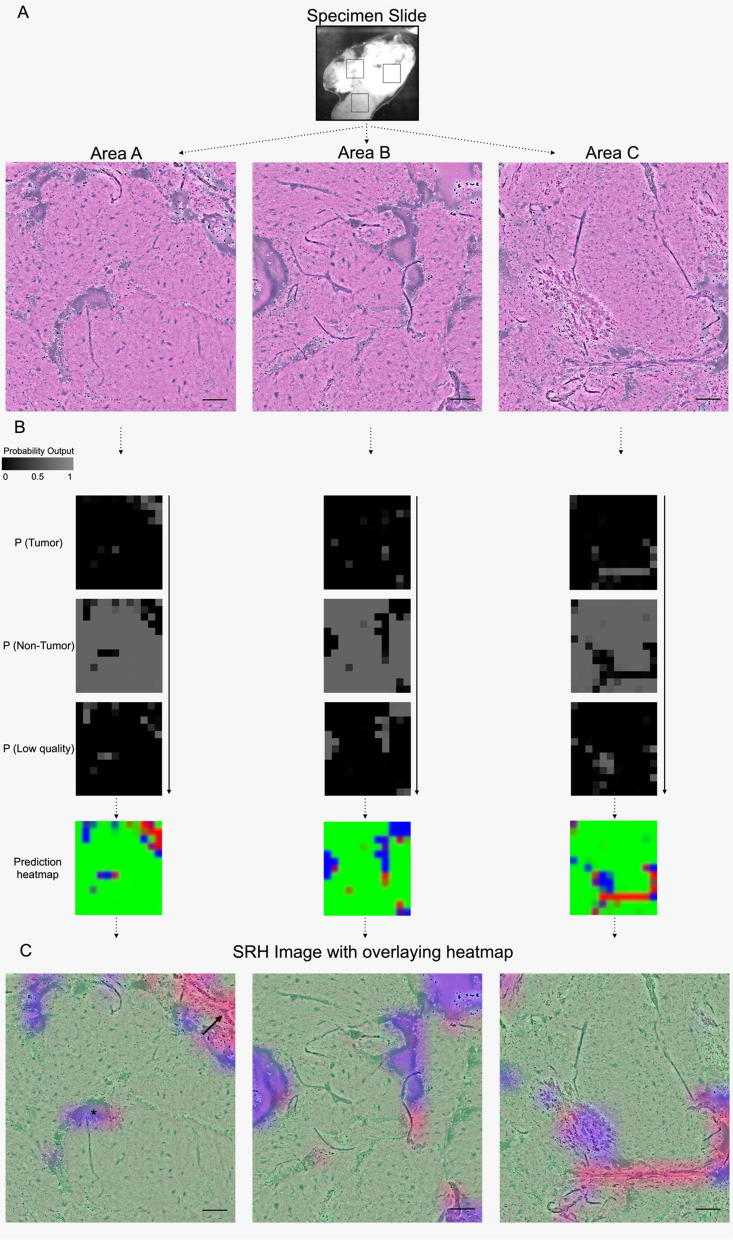
Visual demonstration and internal consistency of the third CNN. **A** Shown is a specimen slide from the surgical approach, close to the tumor margin of a non-small cell lung cancer metastasis, with three random areas (**A**, **B**, **C**). **B** Digital H&E-like images **A**–**C**. **C** Visualization of prediction heatmaps for each output class. **D** Shown are the overlaying heatmaps with the largest and correctly calculated green area part for non-tumorous tissue (white matter). The asterisk and arrow in the middle image demonstrate small non-significant differences at patch size level between random areas. Scale bars = 100 μm

The third CNN, which presented the strongest agreement compared to the neuropathological evaluation, demonstrated an excellent IC in 90.2% (Cα = 0.942) of the specimen slides. Here, the qualitative SRH image analysis with a review of the overlaying heatmap of the CNN showed minor differences in some areas on patch size level, which did not affect the overall prediction output (Further information see Fig. 5 as Additional file 1: Tables S1 and S2).

## Discussion

One of the main challenges in neurosurgery remains to distinguish tumorous tissue safely and efficiently from non-tumorous brain tissue to enable successful stereotactic biopsies and surgically complete resections. In addition, reliable quick histopathological diagnostics are increasingly needed for intraoperative decision-making and gain importance, e.g. for applying local adjuvant treatment options such as chemotherapeutic wafers [11] or intraoperative radiotherapy [12, 13]. The use of conventional rapid frozen section diagnostics with tissue processing and sectioning and white light microscopy by the (neuro)pathologist is the gold standard of care but involves a physical and time-consuming disruption of the surgical workflow [3]. Despite efficiency optimization of the process over years it remains user-dependent, costly- and time-consuming with 20 to 30 min turnaround time, in some cancer centers even longer, and needs high resource requirements due to interdepartmental logistical challenges [3, 14]. Furthermore, destructive, freezing, and compressing artifacts can occur during tissue processing [14]. Lastly, a (neuro)pathology unit is not always available in every surgical tumor center.

As an alternative histopathological assessment pathway the digital SRS microscopy has recently been introduced in the clinical setting as a label- and dye-free microscopic chemical imaging technique for unprocessed tissue specimens [8, 9]. The additive and encouraging use of AI applications with SRH microscopy has already been reported, allowing rapid prediction of tumor entity and glial dignity with encouraging agreement with the neuropathologist's diagnosis [4, 5]. Hollon et al. recently demonstrated an SRH-based CNN to distinguish between predefined tumor entities and dignity [5] but was limited in a qualitative conclusion on whether tumor or no tumor tissue is present. A qualitative assessment of a CNN that distinguishes between tumor and non-tumor has not yet been reported.

We developed three CNNs trained and tested on the same dataset and examined their diagnostic performance, reliability between CNNs, between CNNs and a neuropathologist, and the IC of multiple random areas within a sample.

Using cellular and nuclear morphological features, our trained and deployed CNNs were able to accurately identify and discriminate tumor from non-tumorous brain tissue or judge them as low-quality tissue by analyzing SRH images in a fully automated pipeline.

No significant differences in terms of probability prediction values of the tumor, non-tumor, and low-quality class were found between the three trained CNNs. In this context, previous studies show similar values but did not report detailed analyses of prediction probability values of several CNN models [4, 5, 14, 15]. We were able to demonstrate excellent concordance of the diagnosis prediction between the three CNNs, although some differences in qualitative image inspection were found at the patch size level, which did not negatively affect the overall prediction output of the whole SRH image.

Furthermore, in the reliability analysis between the CNNs and the independent neuropathological evaluation as the gold standard, the third developed CNN demonstrated the highest inter-rater agreement with approximately 90% and showed excellent diagnostic sensitivity and specificity regarding the evaluation of tumor, non-tumor tissue, and low-quality SRH images. It was therefore chosen for further validation and analysis. Other studies showed similar overall accuracy as well as ROC-AUC ranging from approximately 84 to 97% between the CNN- and neuropathological-based SRH image analysis [4, 5, 15, 16]. However, it should be noted that the CNN deployed in these studies had different output classes with different loss functions.

The question has been previously raised whether multiple scans in different areas of the same sample may even increase the diagnostic sensitivity. However, the intra-sample consistency throughout multiple areas has not yet been addressed in previous studies. In that regard, we found an excellent consistency of the applied prediction output between the three different random areas from the corresponding specimen slide of tumor, tumor margin area, or healthy brain tissue. Even if small differences were observed on patch size level, the renormalized summation could anyway result in a consistent diagnosis. In the evaluation of IC, the third trained CNN with the highest agreement in accordance with the neuropathological evaluation also showed an excellent agreement between three random areas within a specimen sample in 90.2%. This suggests that multiple scans within one sample in random areas may be deemed no longer necessary, resulting in an additional intraoperative time-saving.

Despite intraoperative technological advances like neuronavigation, intraoperative MRI-guidance, fluorescence-guided surgery, ultrasound modalities, and recently augmented reality applications [17–23], the surgeon still relies on rapid intraoperative histopathological diagnoses on a qualitative microscopic level for decision-making, as the aforementioned techniques have diverse limitations such as being prone to potential brain shift, limited resolution sensitivity and signal specificity as well as dependent to the varying uptake of fluorescent dyes used [18, 24].

Alternative advanced techniques to our presented AI-based intraoperative optical laser system for qualitative diagnostics are the intraoperative confocal laser endomicroscopy. It provides noninvasive real-time imaging. However, this technique is limited by the need for potential toxic fluorophores for contrast-enhancement and microstructure visualization [25]. Moreover, this technique requires intense user training and merely provides non-static gray scale confocal microscopic images [26–28], which is challenging imaging data to interpret even for trained neuropathologists. In comparison, the CNN-based label-free SRH image acquisition and analysis studied here provides user independently fast feedback, and applicability in the operating room ex-situ and digital images familiarly color-coded like conventional H&E staining. The immediate assessment and connectivity through digital image transfer systems may even provide rapid image and analysis review by the (neuro)pathologist remotely.

Further randomized prospective studies in comparison to conventional histopathological workflows are mandatory for more detailed analysis and validation to establish such a workflow as a potential standard of care on a sustainable basis.

A limitation of our study is that the neuropathological evaluation and, thus, assessment of the ground truth for CNN comparison was performed by only one board-certified neuropathologist. However, all pathological reviews of the SRH images have been done by the same trained independent neuropathology attending with over 20 years of experience minimizing the systematic error. In addition, the diagnosis strategies differed between the CNNs (highest probability prediction value) and the neuropathologist (binary value), which could influence the final comparison and therefore the accuracy of comparison between both. Since the CNN algorithm sums the patches and averages their prediction values for each of the three output classes, rather than providing the highest tumor prediction value of any of the patches, the sensitivity of CNN-based tumor probability diagnosis is, thus, limited in images containing very small tumor infiltrations. That is, in particular, true in cases when single or very few patches predict tumor—equivalent to tumor infiltration in the low micrometer range—while the rest of the SRH image is free of tumor. However, the averaging approach minimizes the misdiagnosis of the sample due to potential artifactual tumor detections in individual patches given the small size of a single patch (14.2 × 13.8 µm) relative to the whole SRH image (1.7 × 1.8 mm). In order to still enable the image reader’s correct interpretation of the CNN results in those cases, SRH image overlaying probability maps are provided in addition and spatially color-code these small tumor areas for a focused look where the CNN has detected tumor.

Additionally, it reports on a large dataset representing the entire spectrum of neurooncological diagnoses.

## Conclusions

In conclusion, CNN-based intraoperative evaluation of SRH images enables label-free tumor detection, irrespective of dignity and entity, in a fully automated pipeline and provides a reproducible alternative pathway for intraoperative decision-making in neurooncological surgery, independent of the conventional histopathologic frozen section examination.

## Supplementary Information

**Additional file 1. Table S1**: Internal consistency analysis and overall agreement of the three residual Convolutional Neural Networks (CNNs). **Table S2**: Comparison of prediction probabilities of the three residual Convolutional Neural Networks (CNN) of the corresponding random areas of the same tumor samples in mean and standard deviation, and Intraclass correlation coefficient (ICC). **Table S3**: Subdivision of predictions of neuropathological evaluation (NP), residual convolutional neural network (CNN), and each interrater agreement (CP) based on the different entities.

## Data Availability

The datasets used and/or analyzed during the current study are available from V.N. on reasonable request.

## Abbreviations

AI: Artificial intelligence
CNN: Convolutional neural network
DL: Deep learning
ICC: Intraclass correlation coefficient
IC: Intrasample consistency
ML: Machine learning
MLA: Machine learning algorithm
SRS: Stimulated Raman scattering
SRH: Stimulated Raman histology
SR: Surgical resection
SB: Stereotactic biopsy
ROC: Receiver operating characteristic
ROC-AUC: Area under the ROC curve
