# Supplementary material for: Novel rapid intraoperative qualitative tumor detection by a residual convolutional neural network using label-free stimulated Raman scattering microscopy
Source: Acta Neuropathol Commun. 2022 Aug 6;10:109. doi: 10.1186/s40478-022-01411-x (PMC9356422; doi:10.1186/s40478-022-01411-x)
Supplement: Supplementary file 1 — Additional file 1. Table S1: Internal consistency analysis and overall agreement of the three residual Convolutional Neural Networks (CNNs). Table S2: Comparison of prediction probabilities of the three residual Convolutional Neural Networks (CNN) of the corresponding random areas of the same tumor samples in mean and standard deviation, and Intraclass correlation coefficient (ICC). Table S3: Subdivision of predictions of neuropathological evaluation (NP), residual convolutional neural network (CNN), and each interrater agreement (CP) based on the different entities. [file 40478_2022_1411_MOESM1_ESM.docx]

**Additional file 1**

**Supplemental Table 1.** Internal consistency analysis and overall agreement of the three residual Convolutional Neural Networks (CNNs).

|  |  |  |  |  |
| --- | --- | --- | --- | --- |
|  |  |  |  |  |
| SRH images (n=402) | |  |  |  |
|  |  |  |  |  |
| Overall Agreement | | 117 slides | 88.6% |  |
|  |  | CNN 1 | CNN 2 | CNN 3 |
| Internal consistency | | 118 (89.4%) | 120 (90.9%) | 119 (90.2%) |
| Cronbach Alpha | | 0.938 | 0.950 | 0.942 |

**Supplemental Table 2**. Comparison of prediction probabilities of the three residual Convolutional Neural Networks (CNN) of the corresponding random areas of the same tumor samples in mean and standard deviation, and Intraclass correlation coefficient (ICC).

|  |  |  |  |  |  |
| --- | --- | --- | --- | --- | --- |
| SRH images (n=402) | |  |  |  |  |
| mean probability | CNN 1 | CNN 2 | CNN 3 | Reliability (ICC) |  |
| (+-SD) |  |  |  |  |  |
|  |  |  |  |  |  |
|  |  |  |  |  |  |
| Random area - part A |  |  |  |  |  |
| tumor | 76.3 (±30.5) | 78.9 (±32.9) | 78.2 (±31.6) | 0.933 (99% CI 0.903-0.954) | |
| non-tumor | 16.8 (±29.6) | 16.1 (±30.9) | 16.0 (±29.3) | 0.971 (99% CI 0.959-0.981) | |
| low quality | 6.9 (±13.2) | 4.9 (±15.3) | 5.7 (±15.6) | 0.806 (99% CI 0.732-0.865) | |
|  |  |  |  |  |  |
| Random area - part B |  |  |  |  |  |
| tumor | 74.8 (±32.2) | 78.0 (±34.0) | 76.7 (±33.4) | 0.957 (99% CI 0.937-0.971) | |
| non-tumor | 18.6 (±31.2) | 18.1 (±32.5) | 18.4 (±31.2) | 0.973 (99% CI 0.961-0.982) | |
| low quality | 6.5 (±12.8) | 3.9 (±12.5) | 4.8 (±14.9) | 0.868 (99% CI 0.809-0.911) | |
|  |  |  |  |  |  |
| Random area - part C |  |  |  |  |  |
| tumor | 72.8 (±34.2) | 76.8 (±35.0) | 76.5 (±34.2) | 0.974 (99% CI 0.954-0.984) | |
| non-tumor | 20.6 (±33.2) | 19.4 (±33.5) | 19.4 (±32.9) | 0.977 (99% CI 0.967-0.985) | |
| low quality | 6.5 (±13.1) | 3.8 (±12.5) | 4.1 (±12.4) | 0.963 (99% CI 0.879-0.984) | |
|  |  |  |  |  |  |

Supplemental Table 3. Subdivision of predictions of neuropathological evaluation (NP), residual convolutional neural network (CNN), and each interrater agreement (CP) based on the different entities.

| Supplemental Table 3. Subdivison of predictions of neuropathological evaluation (NP), residual convolutional neural network (CNN) and each correct prediction (CP). | | | | | | | | | | |  |
| --- | --- | --- | --- | --- | --- | --- | --- | --- | --- | --- | --- |
|  |  |  |  |  |  |  |  |  |  |  |  |
|  |  |  |  |  |  |  |  |  |  |  |  |
|  | SRH images | tumor | | | non-tumor | | | low quality | | |  |
|  |  |  |  |  |  |  |  |  |  |  |  |
| Entities | all (n=402) | NP | CNN | CP (%) | NP | CNN | CP (%) | NP | CNN | CP (%) |  |
|  |  |  |  |  |  |  |  |  |  |  |  |
| Various metastases | 123 | 98 | 101 | 93 (94.9%) | 21 | 19 | 13 (61.9%) | 4 | 3 | 3 (75.0%) |  |
| Malignant glioma | 93 | 75 | 69 | 65 (86.7%) | 15 | 21 | 10 (66.7%) | 3 | 3 | 3 (100%) |  |
| Meningioma | 51 | 49 | 51 | 46 (93.9%) | 2 | 0 | 0 (0%) | 0 | 0 | 0 (0%) |  |
| Diffuse low-grade glioma | 36 | 32 | 31 | 31 (96.9%) | 4 | 5 | 4 (100%) | 0 | 0 | 0 (0%) |  |
| Pituitary adenoma | 18 | 17 | 18 | 17 (100%) | 0 | 0 | 0 (0%) | 1 | 0 | 0 (0%) |  |
| Ependymoma | 12 | 12 | 11 | 11 (91.7%) | 0 | 0 | 0 (0%) | 0 | 1 | 0 (0%) |  |
| Lymphoma | 12 | 12 | 12 | 12 (100%) | 0 | 0 | 0 (0%) | 0 | 0 | 0 (0%) |  |
| Craniopharyngioma | 9 | 9 | 8 | 8 (88.9%) | 0 | 1 | 0 (0%) | 0 | 0 | 0 (0%) |  |
| Pilocytic astrocytoma | 6 | 4 | 6 | 4 (100%) | 2 | 0 | 0 (0%) | 0 | 0 | 0 (0%) |  |
| Schwannoma | 6 | 5 | 5 | 5 (100%) | 1 | 1 | 1 (100%) | 0 | 0 | 0 (0%) |  |
| Hemangioblastoma | 6 | 3 | 4 | 3 (100%) | 3 | 2 | 2 (66.6%) | 0 | 0 | 0 (0%) |  |
| Medulloblastoma | 4 | 4 | 4 | 4 (100%) | 0 | 0 | 0 (0%) | 0 | 0 | 0 (0%) |  |
| Ganglioneurinoma | 3 | 3 | 3 | 3 (100%) | 0 | 0 | 0 (0%) | 0 | 0 | 0 (0%) |  |
| Rathke cleft cyst | 3 | 3 | 3 | 3 (100%) | 0 | 0 | 0 (0%) | 0 | 0 | 0 (0%) |  |
| Gliosis | 3 | 0 | 0 | 0 (0%) | 2 | 3 | 2 (100%) | 1 | 0 | 0 (0%) |  |
| Negative control (approach) | 17 | 0 | 0 | 0 (0%) | 17 | 17 | 17 (100%) | 0 | 0 | 0 (0%) |  |
| In total | 402 | 326 | 326 | 305 (95.0%) | 67 | 69 | 49 (73.1%) | 9 | 7 | 6 (66.7%) |  |
